# Treatment of parotid gland oncocytoma: a case report from a radiation oncologist’s perspective

**DOI:** 10.3389/fonc.2026.1849527

**Published:** 2026-06-01

**Authors:** Kemeng Zhao, Feifei Hu, Yong Rui Bai

**Affiliations:** SDepartment of Radiation Oncology, Renji Hospital, Shanghai Jiao Tong University School of Medicine, Shanghai, China

**Keywords:** case report, oncocytoma, parotid, radiotherapy, salivary gland

## Abstract

**Background:**

Parotid oncocytoma is a rare benign neoplasm for which surgical excision remains the standard treatment. Although the recurrence rate is generally low, it increases significantly in cases with multifocal lesions or incomplete surgical excision. In such scenarios, postoperative radiotherapy (RT) has been shown to improve local control, reduce recurrence rates, and minimize the need for reoperation. This report presents a representative case of parotid oncocytoma, with a focused discussion on therapeutic decision-making from a radiation oncologist’s perspective.

**Case report:**

We report the case of a 61-year-old man who underwent left superficial parotidectomy for a firm mass located below his left ear. Postoperative pathological examination confirmed parotid oncocytoma. Contrast-enhanced computed tomography (CT) revealed an enhancing nodule in the deep portion of the left parotid gland, measuring 1.6 cm in maximum diameter. Following multidisciplinary team (MDT) discussion, postoperative radiotherapy was recommended to avoid potential facial nerve injury associated with repeat parotid surgery.

**Conclusion:**

This case illustrates the use of postoperative adjuvant radiotherapy for left parotid oncocytoma. Although parotid oncocytoma is benign, adjuvant radiotherapy may be considered in selected cases with a high risk of recurrence.

## Introduction

Oncocytoma, also known as oxyphilic adenoma or mitochondrioma, is a rare benign epithelial neoplasm. Salivary gland tumors account for approximately 3% of all head and neck neoplasms, with oncocytomas representing only 0.1% to 1.5% of these cases (1, 2). Most salivary gland oncocytomas occur in the parotid gland; they rarely arise in the larynx, tonsillar fossa, and lacrimal gland (2). This tumor can also originate in other organs, such as the kidney, adrenal, thyroid, and pituitary glands (2).

Epidemiologic data indicate that oncocytomas are most frequently diagnosed in individuals aged 70–80 years (3). Clinically, parotid gland oncocytomas typically present as solitary, nonspecific masses. Patients often report persistent swelling lasting from weeks to years; these tumors are usually painless and mobile on palpation (1).

The World Health Organization (WHO) classifies salivary gland oncocytic neoplasms into three categories: oncocytosis, oncocytoma, and oncocytic carcinoma. Oncocytoma is the most common type and is histologically characterized by monotonous sheets of epithelial cells (oncocytes) with a central scar (2). Although oncocytes may be present in conditions ranging from hyperplasia to malignancy, oncocytomas are defined by the pervasive presence of these distinctive cells throughout the lesion (4). The risk factors for oncocytoma remain unknown, however, approximately 20% of cases have been associated with prior radiation exposure at least five years before diagnosis (2). While oncocytomas are classically characterized as slow–growing and asymptomatic neoplasms, some cases exhibit more aggressive behavior such as invasion into the surrounding connective tissue capsule. Although distant metastasis or recurrence have not been observed, it implies malignant transformation may occur in such patients (5). Overall, the prognosis for oncocytomas is favorable, with a reported recurrence rate of 2.7% (6) However, incomplete surgical resection significantly increases the risk of local recurrence (7).

## Case description

A 61–year–old male presented to our department nearly two months after undergoing left superficial parotidectomy for a parotid gland oncocytoma. The patient first noticed a mass below his left ear one year prior. Initially, the mass was firm, non–tender, and well–circumscribed, and no specific intervention was performed. Following the onset of localized pain in early 2025, the patient initially sought care at a local hospital and had an ultrasound imaging which revealed a hypoechoic area in the left parotid gland measuring approximately 28 mm × 20 mm, with regular margins and uneven internal echoes. Based on these findings, the patient underwent a left parotidectomy on January 21, 2025, and a specimen measuring roughly 2 cm × 3 cm was excised.

**Figure 1:**
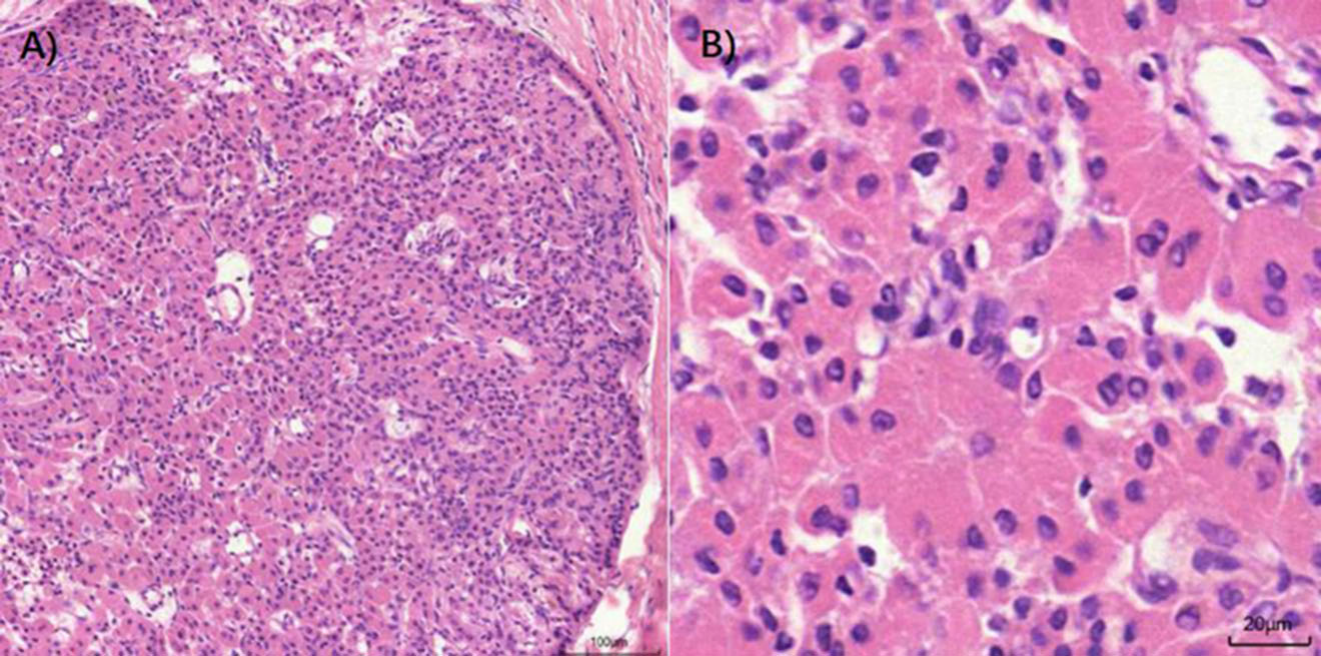
Hematoxylin and eosin (HE) stain. **(A)** Low-power view shows a solid mass with a lobular border, composed of polygonal oncocytic cells; **(B)** High-power view reveals large groups of polygonal cells with clear and eosinophilic granular cytoplasm, large round nuclei, and prominent nucleoli, consistent with oncocytoma.

Histopathological examination confirmed parotid oncocytoma, showing focal eosinophilic cytoplasmic alterations in acinar cells, unencapsulated nodules, and a multifocal nodular growth pattern (**Figure 1**). Immunohistochemical staining revealed diffuse AE1/AE3 positivity, focal p40/p63 expression, strong CK7 reactivity, partial EMA staining, and a Ki-67 proliferation index of 10% in hotspot regions.

**Figure 2:**
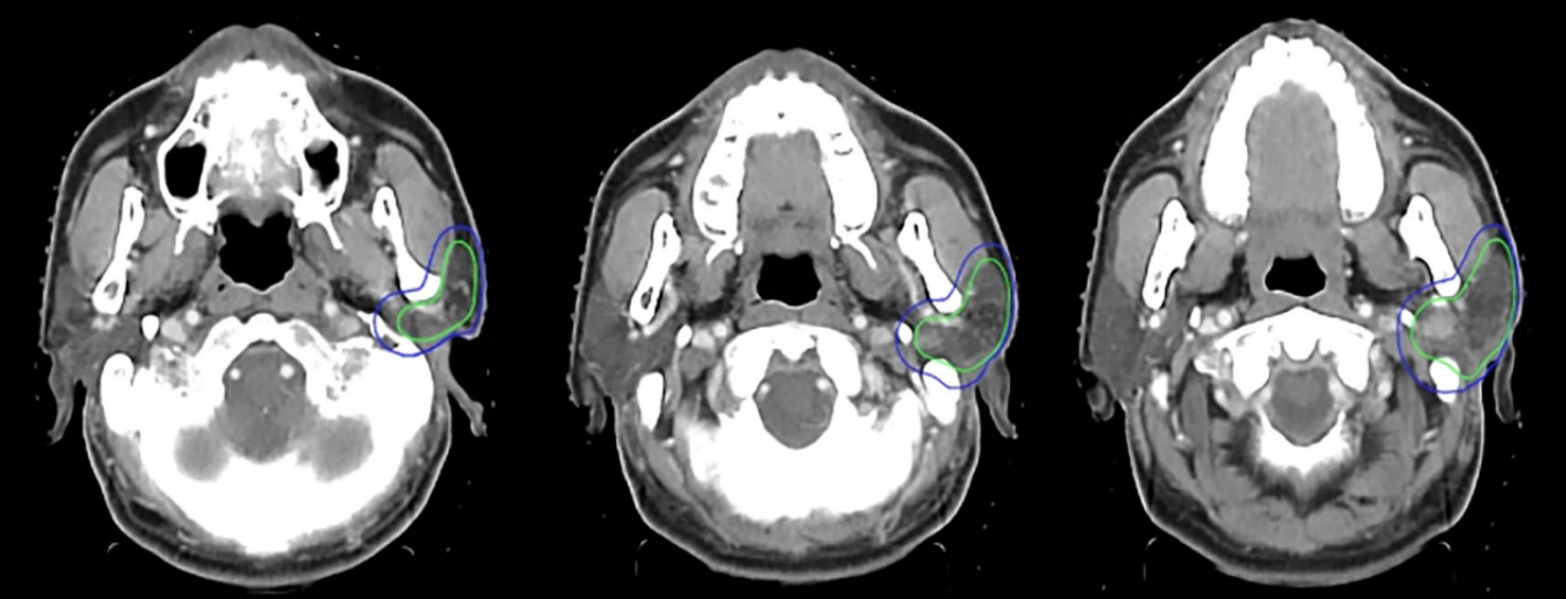
Axial contrast-enhanced planning CT (blue line: PTV; green line: CTV).

**Figure 3:**
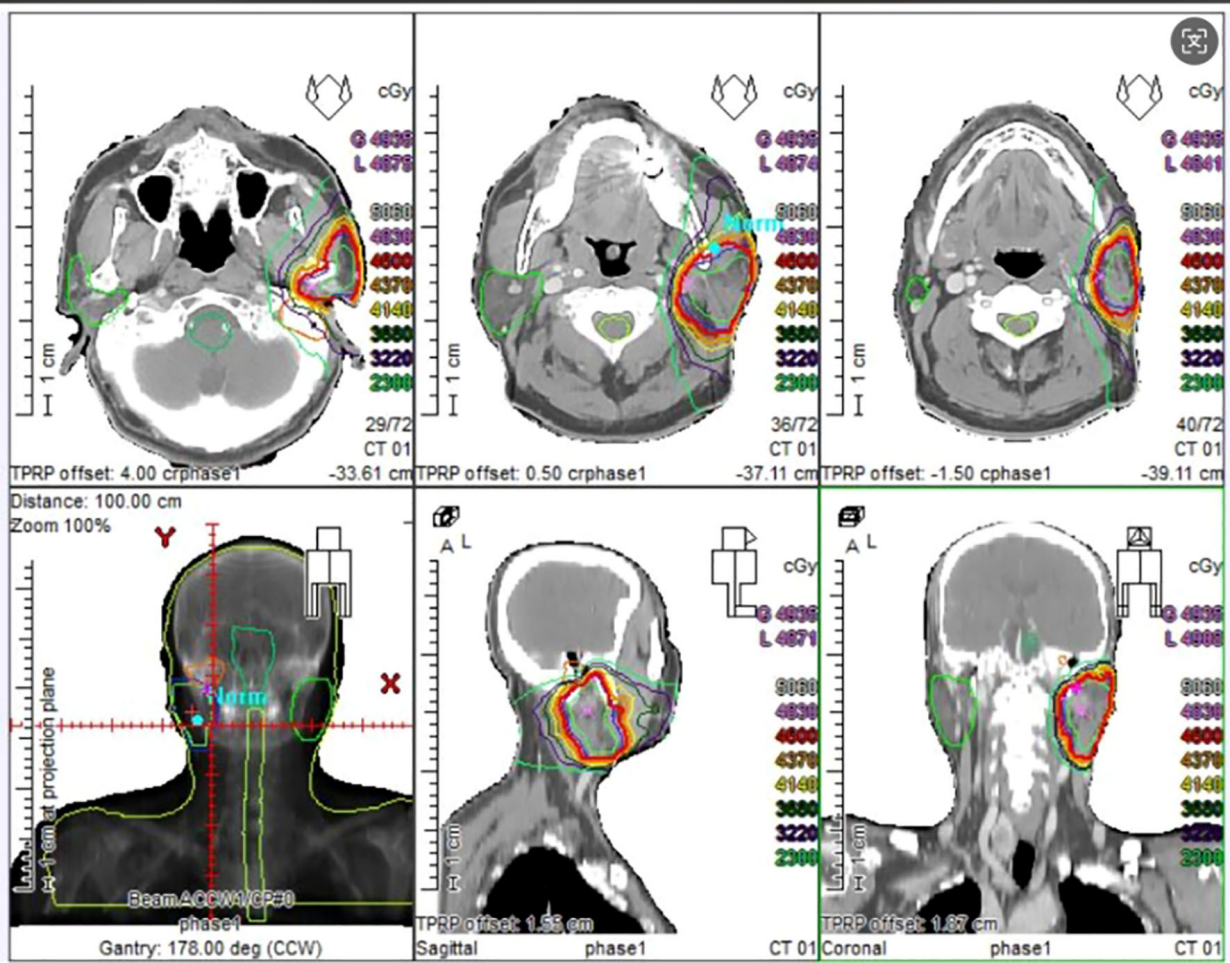
Isodose distribution of postoperative radiotherapy (red line: 100% of prescribed dose; green line: 50% of prescribed dose).

The patient was evaluated in the Radiotherapy Department on March 20, 2025. Physical examination at the outpatient clinic revealed a well-healed, 6-cm curvilinear surgical scar in the left postauricular region. No palpable cervical or supraclavicular lymphadenopathy was noted, and no distinct neck masses were identified. And he had an unremarkable past medical history with no underlying conditions, denied any tobacco or alcohol use, and had no family history of similar malignancies. Contrast-enhanced CT simulation demonstrated an enhancing nodule in the deep portion of the left parotid gland, with a maximum diameter of 1.6 cm. Combined with the radiological appearance and the biological characteristic of oncocytoma, we suspect the enhancing nodule to be a residual or multifocal oncocytoma lesion. After MDT discussion, postoperative adjuvant radiotherapy to the residual parotid gland area was recommended to avoid the potential facial nerve injury associated with repeat surgery. The clinical target volume (CTV) included the left parotid gland area and the residual deep parotid nodule. The planning target volume (PTV) was defined by a 3 mm expansion around the CTV, as shown in **Figure 2**. A total dose of 46 Gy in 23 fractions was prescribed (**Figure 3**). This prescribed dose fractionation regimen is aimed at eradiating the residual node and potential subclinical lesions to maximum local control while protecting adjacent normal tissues from severe late toxicities.

The patient successfully completed the course of radiotherapy. During treatment, the patient developed mild radiotherapy-induced oral mucositis (RTOG Grade 1), and he did not receive specific medical intervention. Following the completion of therapy, the patient reported a gradual and spontaneous relief of related symptoms.

**Figure 4:**
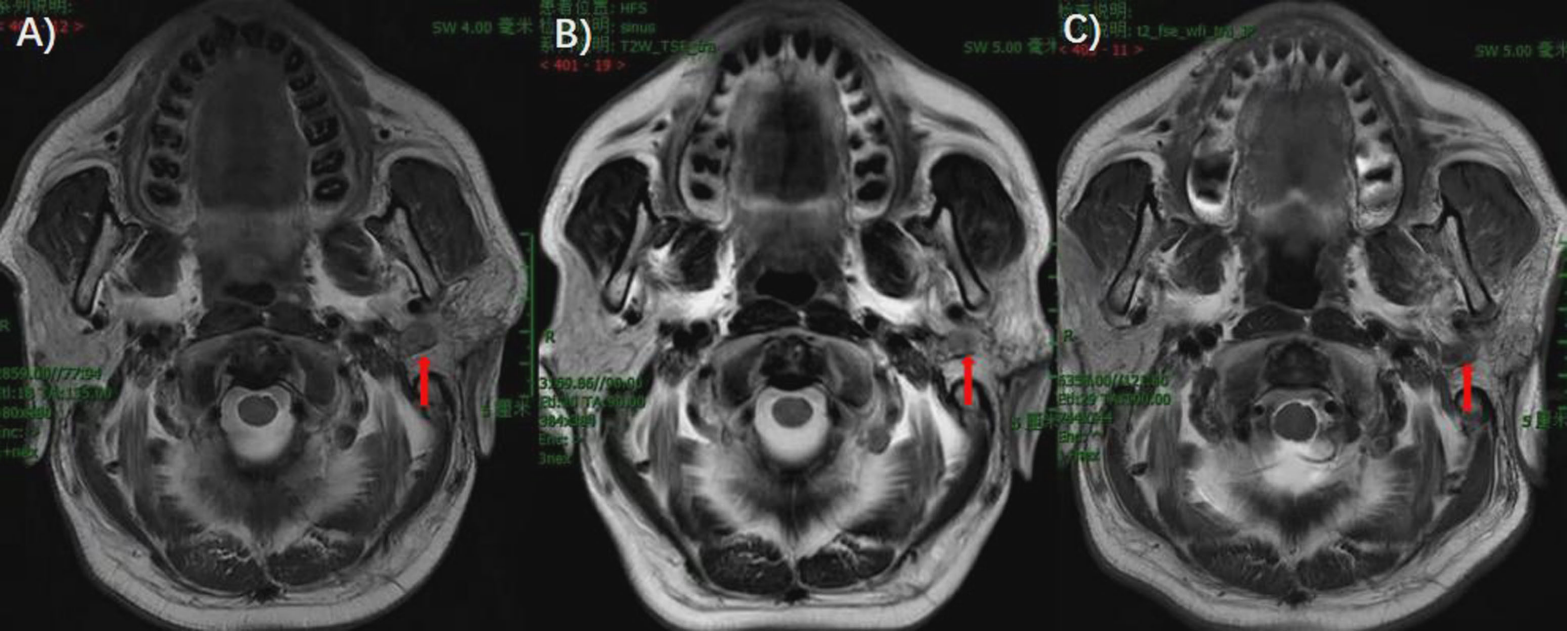
T2-weighted MRI follow-up findings. **(A)** During radiotherapy (after 13 fractions); **(B)** One month post-radiotherapy; **(C)** Three months post-radiotherapy.

The patient underwent follow-up MRI regularly. Compared with the initial scan obtained during radiotherapy, subsequent images showed a reduction in the size of the left parotid gland deep lobe nodule and relief of edema in the adjacent muscle and subcutaneous soft tissue (**Figure 4**).

## Discussion

Oncocytoma is a benign epithelial tumor characterized by the presence of oncocytes—cells exhibiting eosinophilic granular cytoplasm due to abundant mitochondria (8). Given that oncocytic metaplasia and oncocytomas predominantly occur in older adults, oncocytes were historically regarded as a senescent or degenerative cellular state (9). Current evidence, however, suggests that oncocytic transformation represents a response to dysregulated cellular metabolism and disturbances in mitochondrial enzyme organization (10). Oncocytes are believed to arise from metaplastic transformation of ductal or acinar epithelial cells within salivary glands (8). Immunohistochemical analyses supporting this origin show expression of ductal epithelial markers such as CK7, CK8, and CK19 (11).

MRI and CT are the primary imaging modalities for evaluating parotid gland tumors (1). Parotid oncocytomas typically present on CT as a well-defined, homogeneously enhancing mass (12). However, up to 50% of oncocytomas exhibit heterogeneous enhancement, potentially mimicking malignancy (7). On MRI findings, they frequently appear isointense to adjacent parotid tissue on both T1- and T2-weighted sequences, though atypical signal intensities can occur (13). Furthermore, while ultrasound-guided fine-needle aspiration cytology (FNAC) generally provides high diagnostic accuracy for parotid neoplasms (12), it struggles to challenge definitively identify oncocytomas due to the presence of oncocytes in a spectrum of salivary gland pathologies, ranging from benign hyperplasia to malignancy. Furthermore, limited sampling of focal lesions and overlapping cytomorphological features often hinder definitive diagnosis via FNAC (14). A large series by Capone et al. reported a sensitivity of only 29% for FNAC in detecting oncocytoma (15). Thus, reliance on FNAC alone, without correlation with clinical and imaging data, is unjustified and potentially misleading. Recently, F-18 FDG PET/CT has shown promise in distinguishing salivary gland malignancies; however, due to limitations, it is not always preferred, even in cases with biopsy findings suggestive of malignancy (11). Notably, surgical resection via parotidectomy remains essential for both diagnosis and treatment, with histopathological analysis serving as the gold standard for definitive diagnosis (14).

Surgical excision—via radical or superficial parotidectomy—is the mainstay of treatment for parotid oncocytoma. The extent of surgery is guided by preoperative clinical and radiological assessments (e.g., CT, MRI) and intraoperative findings (8). Postoperative radiotherapy is an effective adjuvant treatment for most of parotid malignancies, particularly in cases of advanced-stage disease (T3/T4), intermediate- or high-grade histology, close or positive surgical margins, or lymph node metastasis. Additional adverse features warranting adjuvant radiotherapy include bone invasion and perineural or vascular involvement. Radiotherapy may also be employed as definitive treatment for unresectable parotid carcinomas (16). However, post-operative radiotherapy is not commonly recommended for the treatment of salivary gland benign tumors according to NCCN guidelines (17). In over 95% of benign salivary gland tumors, surgical resection with negative margins achieves local control without adjuvant radiotherapy, given its limited therapeutic benefit and potential morbidity (18). Besides, oncocytes have the nature of radioresistance (19). Thus, complete surgical excision is typically curative for salivary gland oncocytoma.

In this case, the patient underwent left parotid gland resection, with histopathological confirmation of parotid oncocytoma. And postoperative imaging revealed a suspected residual tumor. Although local recurrence of oncocytoma after complete surgical excision is uncommon, the risk increases to approximately 20–30% in cases of incomplete resection or multifocal disease (8, 12). Considering this, the patient may be faced with the potential facial nerve injury that reoperation results in.

Cases describing postoperative adjuvant radiotherapy for parotid oncocytomas are exceedingly rare. However, it is reported that radiotherapy still exerts great influence in the field of benign salivary gland tumors. Some evidence showed that adjuvant radiotherapy can improve local control in cases of multinodular recurrence or metastasizing pleomorphic adenoma (20–22), which establish a precedent and provide a reference for the application of radiotherapy to other benign yet troublesome salivary gland tumors. Similarly, oncocytoma can present as a multifocal lesion and recur if not completely resected. Based on these findings, after MDT discussion, combining the needs of the patient, postoperative radiotherapy was administered to mitigate the risk of local recurrence. The CTV included the left parotid region and the residual deep parotid nodule. A prescribed dose of 46 Gy in 23 fractions was delivered. Although the treatment response has been favorable to date, the patient has not yet completed long-term follow-up.

It should be noted that no standardized global diagnostic or therapeutic guidelines exist for oncocytoma currently and surgical intervention remains the most frequent management strategy, with postoperative adjuvant therapies not yet definitively established. Nevertheless, recent evidence suggests that stereotactic body radiation therapy (SBRT) may offer an alternative for local control in non-surgical renal oncocytoma patients (23). Therefore, further studies and long-term follow-up are still needed to determine the true value of radiotherapy in such scenarios. The prognosis of oncocytomas is favorable; although rare, malignant transformation into oncocytic carcinoma has been reported (24). Therefore, long-term postoperative follow-up is strongly recommended, with surveillance imaging (preferably MRI) at 12 and 24 months after treatment. This protocol aligns with the recognized recurrence pattern of head and neck malignancies, which predominantly manifest within the first two years (8, 25). Recent MRI (**Supplement1**) demonstrates a reduction in lesion size compared to previous imaging, with the disease remaining stable. These findings support the role of radiotherapy as an effective adjuvant modality in the postoperative management of high-risk parotid gland oncocytoma.

## Patient perspective

When my surgery doctor recommended postoperative adjuvant radiotherapy, I initially had concerns about the potential side effects. However, after thoroughly discussing the benefits with the radiotherapy team, I fully trusted their decision and felt confident that this was the best approach to reduce the risk of recurrence. To my relief, the actual treatment process was much smoother than I had anticipated. While I did experience some mild dry mouth during the course, the side effects were entirely manageable. They did not significantly interfere with my swallowing, eating habits, or daily activities. My overall quality of life remained well-preserved throughout the whole treatment course. Looking back, I am highly satisfied with the radiotherapy process and deeply grateful for the comprehensive care I received.

## Conclusion

We present a case of left parotid gland oncocytoma treated with postoperative adjuvant radiotherapy. Although parotid oncocytoma is inherently benign and radical excision is the primary treatment, adjuvant therapy is generally unnecessary. However, in cases with incomplete resection or multifocal lesions, the recurrence rate is higher. To mitigate recurrence risks and avoid potential iatrogenic facial nerve injury due to reoperation, for the benefit of the patient, empirical adjuvant radiotherapy may be considered. MRI surveillance at 12 and 24 months postoperatively is strongly recommended, as head and neck malignancies are most likely to recur within the first two years after surgery.

## Data Availability

The raw data supporting the conclusions of this article will be made available by the authors, without undue reservation.
