# Supplementary material for: Treatment of parotid gland oncocytoma: a case report from a radiation oncologist’s perspective
Source: Front Oncol. 2026 Jun 1;16:1849527. doi: 10.3389/fonc.2026.1849527 (PMC13265275; doi:10.3389/fonc.2026.1849527)
Supplement: Supplementary file 1 [file Table1.docx]

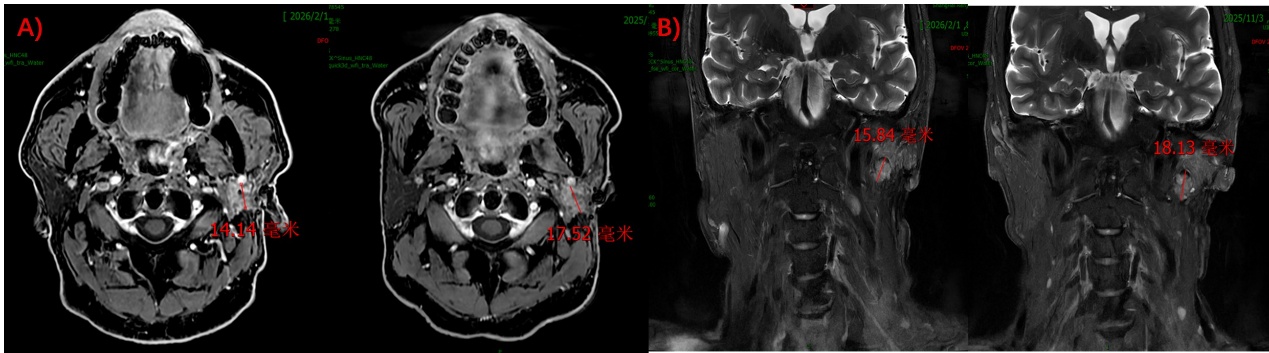
 **Supplement1** MRI follow-up images in 6 months and 9 months after radiotherapy. A) Comparison between T1 MRI images in transverse section; B) Comparison between T2 MRI images in coronal section.
